# A *TRPV4* mutation caused Charcot-Marie-Tooth disease type 2C with scapuloperoneal muscular atrophy overlap syndrome and scapuloperoneal spinal muscular atrophy in one family: a case report and literature review

**DOI:** 10.1186/s12883-023-03260-0

**Published:** 2023-06-30

**Authors:** Haofeng Chen, Chong Sun, Yongsheng Zheng, Junxiong Yin, Mingshi Gao, Chongbo Zhao, Jie Lin

**Affiliations:** 1 Department of Neurology, Huashan Hospital, Fudan University, 12 Middle Wulumuqi Rd, Shanghai, 200040 China; 2 National Center for Neurological Diseases, 12 Middle Wulumuqi Rd, Shanghai, 200040 China; 3 Huashan Rare Disease Center, Huashan Hospital Fudan University, 12 Middle Wulumuqi Rd, Shanghai, 200040 China; 4 Department of Pathology, Huashan Hospital, Fudan University, 12 Middle Wulumuqi Rd, Shanghai, 200040 China

**Keywords:** CMT2C, p.R316C mutation, SPSMA, Sural nerve biopsy, TRPV4

## Abstract

**Background:**

Charcot-Marie-Tooth disease 2C (CMT2C) and scapuloperoneal spinal muscular atrophy (SPSMA) are different clinical phenotypes of *TRPV4* mutation. The mutation of p.R316C has been reported to cause CMT2C and SPSMA separately.

**Case presentation:**

Here, we reported a Chinese family harboring the same p.R316C variant, but with an overlap syndrome and different clinical manifestations. A 58-year-old man presented with severe scapula muscle atrophy, resulting in sloping shoulders. He also exhibited distinct muscle atrophy in his four limbs, particularly in the lower limbs. The sural nerve biopsy revealed severe loss of myelinated nerve fibers with scattered regenerating clusters and pseudo-onion bulbs. Nerve conduction study showed axon damage in both motor and sensory nerves. Sensory nerve action potentials could not be evoked in bilateral sural or superficial peroneal nerves. He was diagnosed with Charcot-Marie-Tooth disease type 2C and scapuloperoneal muscular atrophy overlap syndrome, whereas his 27-year-old son was born with clubfoot and clinodactyly. Electromyogram examination indicated chronic neurogenic changes and anterior horn cells involvement. Although there was no obvious weakness or sensory symptoms, early SPSMA could be considered for him.

**Conclusions:**

A literature review of the clinical characteristics in CMT2C and SPSMA patients with *TRPV4* mutation suggested that our case was distinct due to the overlap syndrome and phenotype variation. Altogether, this case broadened the phenotype spectrum and provided the nerve biopsy pathological details of TRPV4-related neuropathies.

**Supplementary Information:**

The online version contains supplementary material available at 10.1186/s12883-023-03260-0.

## Background

Transient receptor potential vanilloid type 4 (TRPV4) is a nonselective Ca^2+^ permeable cation channel mainly involved in the regulation of systemic osmotic pressure. The TRPV4 protein is composed of six transmembrane domains, a characteristic N-terminal ankyrin repeat domain (ARD) and C-terminal intracellular tails [1]. The central cation conductivity pore is formed by transmembrane domains 5, 6 and the interconnecting loop. Four subunits assemble to form the functional cation channel [2]. The *TRPV4* gene is located on 12q24.11. Mutations in this gene have been identified in skeletal dysplasia, arthropathy and neuromuscular disorders. Different sites of mutation from the N- to C-terminus can lead to diverse clinical manifestations [3]. They are particularly associated with a broad spectrum of neuropathies like scapuloperoneal spinal muscular atrophy (SPSMA), congenital distal spinal muscular atrophy (CDSMA), distal hereditary motor neuropathy (dHMN) and Charcot-Marie-Tooth disease type 2C (CMT2C) [4–8]. Clinically, the phenotypes of neuropathy can vary within the same family with identical mutations. These different phenotypes sometimes may occasionally overlap in one person [9]. In this report, we reported a family harboring the TRPV4 p.R316C mutation manifesting a distinct clinical phenotype between the father and his son. The father had CMT2C and scapuloperoneal muscular atrophy overlap syndrome, while his son had SPSMA. Furthermore, we performed a literature review of the clinical characteristic of CMT2C and SPSMA.

## Case presentation

### Patient 1

This patient was a 58-year-old male born to non-consanguineous parents after an uneventful pregnancy and delivery. He presented with difficulty in playing sports since childhood but with normal developmental milestones. At the age of 48, he began to experience weakness in his four limbs without any obvious cause and had difficulty holding objects and walking upstairs. Starting from the age of 55, he experienced numbness in both hands and feet accompanied by the feeling of wearing gloves and socks. The limb muscle weakness progressively aggravated with atrophy. He had to walk with external help and made a significant effort in holding chopsticks and writing. He has been diagnosed with diabetes for more than twenty years.

**Fig. 1:**
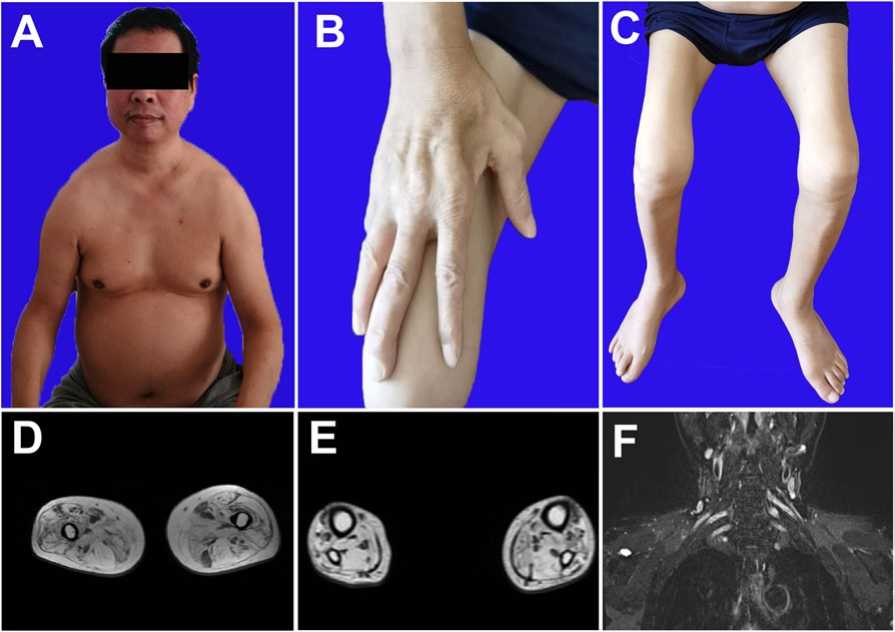
Clinical and MRI findings of patient 1. The patient exhibited scapular muscle atrophy, resulting in sloping shoulders (**A**), as well as significant atrophy of hand intrinsic muscles (**B**) and calf muscles (**C**). MRI revealed atrophy of the thigh (**D**) and calf (**E**) muscles with concurrent fat infiltration. The brachial plexus was hypertrophy and hyperintense (**F**)

The neurologic exam found severe scapula muscle atrophy with sloping shoulders (Fig. 1A). Distinct muscle atrophy was also present in the thenar (Fig. 1B) and the lower limbs (Fig. 1C). Muscle strength was bilaterally 4/5 in elbow extension, 2/5 in finger extension, 2/5 in hip extension and 0/5 in ankle dorsiflexion. Deep tendon reflexes were absent in both the upper and lower extremities without the Babinski sign. Pinprick and vibration sensations were absent in the feet and moderately reduced in the hands. The pyramidal sign was negative. The hearing was normal and there were no findings of hoarseness, pes cavus or scoliosis.

Laboratory testing showed that his creatine kinase was 56U/L, within the normal range. His glucose level was slightly elevated at 7.1 mmol/L and his glycated hemoglobin was 7.1% (normal below 5.7%). Echocardiography manifested a thick interventricular septum with normal contraction function. Magnetic resonance imaging (MRI) indicated mild atrophy of bilateral thigh muscles. Both quadriceps and biceps femoris muscles were involved, while the semitendinosus muscle was partially spared (Fig. 1D). The calf muscle was also atrophic with fat infiltration (Fig. 1E). The brachial plexus was symmetrically hypertrophy and hyperintense (Fig. 1F). Electrophysiology presented that the amplitudes of compound muscle action potentials (CMAPs) were reduced in ulnar nerves and were absent in bilateral peroneal nerves. Sensory nerve action potentials (SNAPs) were not evoked in the right median, ulnar, sural or superficial peroneal nerves. Electromyogram (EMG) showed fibrillation and positive sharp waves in partial muscles. Increased duration and large amplitude of motor unit potentials (MUPs) were found in bilateral calf muscles.

**Fig. 2:**
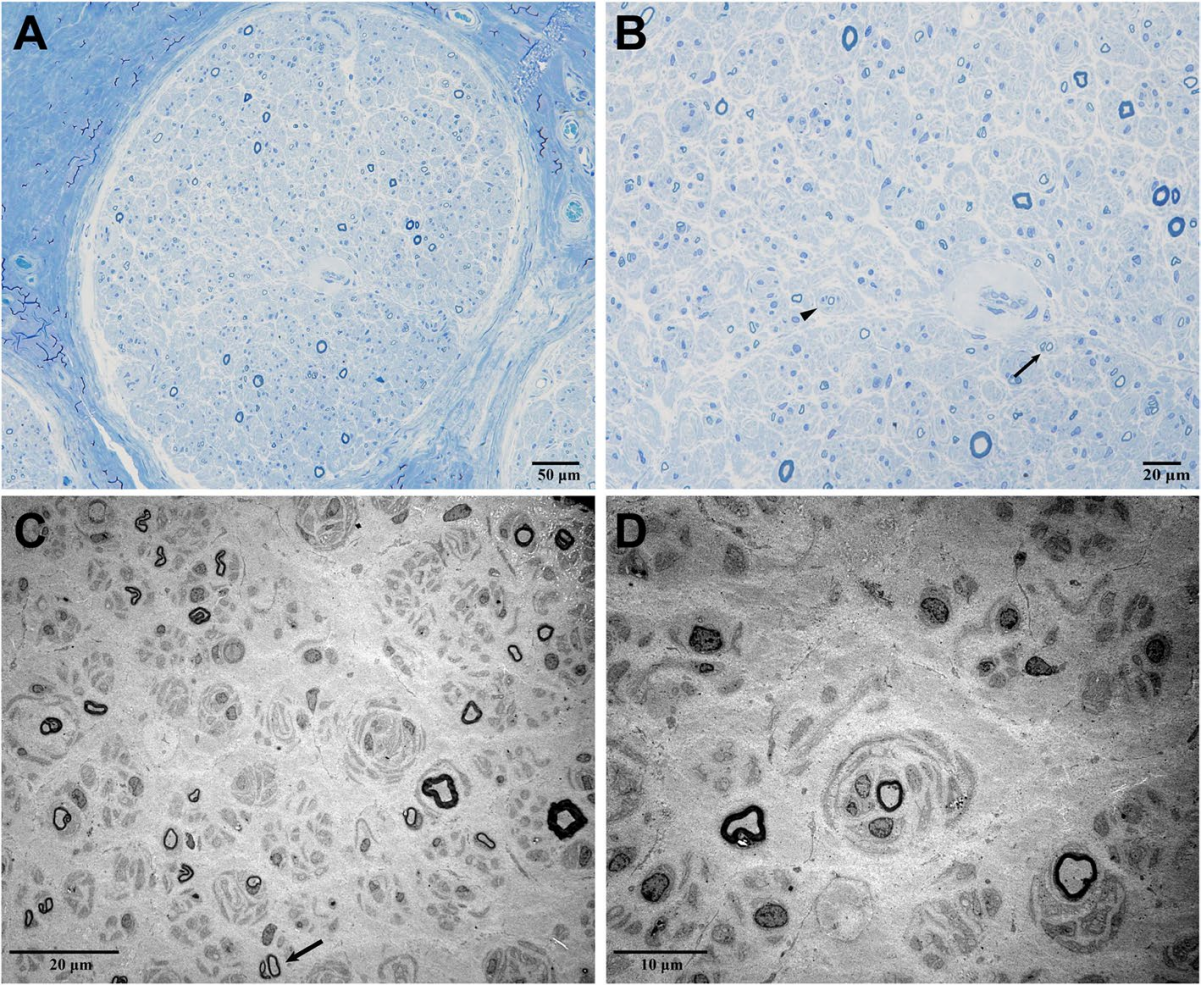
Sural nerve pathology of patient 1. Toluidine-stained semithin sections showed severe decreased density of myelinated fibers (**A**). Regenerating clusters (arrow), pseudo-onion bulbs (arrowhead) can be seen (**B**). Ultrastructure examination also showed regenerating clusters (**C**, arrow) and numerous unmyelinated collateral sprouts forming a pseudo-onion bulb (**D**)

Sural nerve biopsy was performed on this patient. The semi-thin section showed a severe reduction in the density of myelinated fibers (Fig. 2A). The scattered pseudo-onion bulbs formed by regenerating clusters were visible, which were probably a reflection of axonal regeneration (Fig. 2B). Immunohistochemical staining was negative. Ultrastructural examination represented denervated Schwann cell bands, scattered myelinated axons, and numerous unmyelinated fibers regeneration (Fig. 2C). There were collections of concentric Schwann cell processes that enclosed unmyelinated axons (Fig. 2D). More or less Schwann cells sprouted within the fields.

**Fig. 3:**
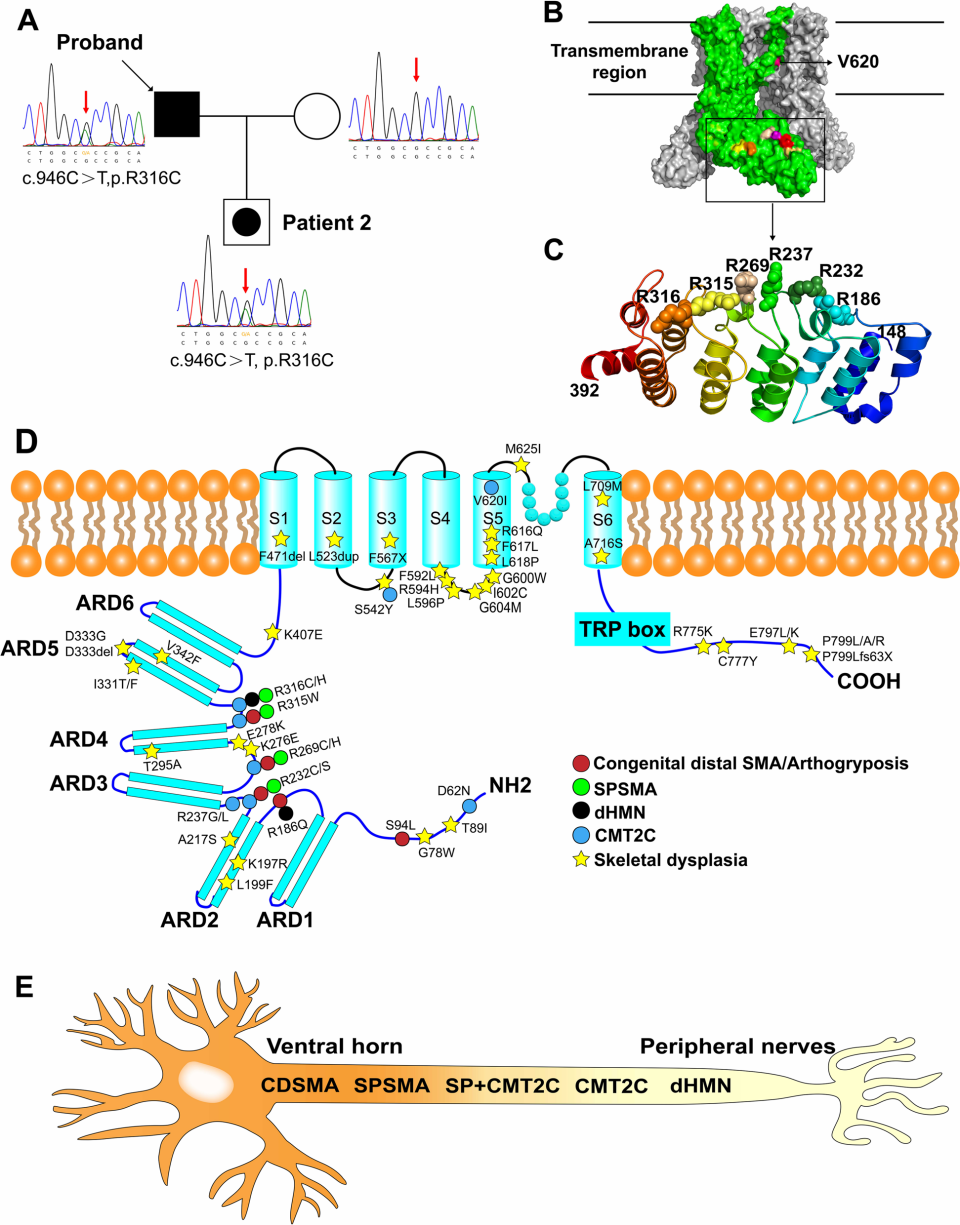
Pedigree of this family and the chromatograms of each member (**A**). Crystal structure model of Human TRPV4. One subunit was shown in green and the rest in grey. Mutation sites related to CMT2C and SPSMA were marked. Only the V620 was located in the transmembrane region. The other sites were located in the ARD region (**B**). The mutation sites in the ribbon diagram of the ARD region (residues 148–392) were labeled. All of these sites were in the connecting finger loop between the ankyrin repeats (**C**). Schematic illustration of the TRPV4 protein domains is accompanied with the location of specific phenotype of neuropathy and skeletal dysplasia respectively (**D**). Skeletal dysplasia was located in the helices, the convex face of TRPV4 ARD, where neuropathy-related mutations have not been described. The phenotypes of TRPV4-related neuropathies were a spectrum from the ventral horn cell to the distal peripheral nerve (**E**). Black filled symbol = affected; empty symbol = unaffected; empty symbol with black dot = mildly affected mutation carrier in clinical

Genetic analysis revealed that this patient had a heterozygous c.946C > T mutation in the *TRPV4* gene, resulting in amino acid substitutions of arginine to leucine (p.R316C). This mutation was absent in his wife but was heterozygous in his son (Fig. 3A).

### Patient 2

This 27-year-old patient was the son of patient 1. He was born with clubfoot and underwent corrective surgery at the age of seven. Clinodactyly was noted in his left foot, while pes cavus was not detected. He complained of thigh muscles twitching after exertions. However, there was no apparent weakness. He also denied any sensory symptoms and extra nervous manifestations. In the nerve conduction study, the CMAPs of the right common fibular nerve were not evoked, while the sensory nerves were normal. The EMG examination indicated chronic neurogenic changes, and muscles in his limbs and trapezius muscle were affected with increased duration and large amplitude MUPs. The involvement of anterior horn cells was therefore taken into consideration.

## Discussion and conclusions

In this study, we reported a Chinese family with diverse phenotypes of neuropathy caused by c.946C > T (p.R316C) mutation in *TRPV4* gene. Patient 1 was diagnosed with CMT2C and scapulopeoneal muscular atrophy overlap syndrome. However, his 27-year-old son (Patient 2) who presented chronic neurogenic damage in EMG without obvious muscle weakness was considered early SPSMA. A comparison of clinical and electrophysiological details between Patient 1 and Patient 2 is presented in Table 1.

**Table 1:** Clinical and electrophysical details in this family

	**Patient 1**	**Patient 2**
**Muscle strengths**		
Eyes closing	5,5	5,5
Shoulder abduction	4,4	5,5
Elbow flexion	4,4	5,5
Wrist extension	2,2	5,5
Hip flexion	2,2	5,5
Knee extension	2,2	5,5
Foot dorsiflexion	0,0	5-,5-
**NCS (Right Motor nerve)**		
Median CV(> 50.0 m/s)	50.1	55.4
Median Amp(> 4.8mv)	1.75	5.3
Ulnar CV(> 51.0 m/s)	59.8	54.7
Ulnar Amp (> 5.5mv)	0.6	6.9
Peroneal CV(> 39.8 m/s)	NP	NP
Peroneal Amp (> 2.3 mv)	NP	NP
**NCS (Right Sensory nerve)**		
Median CV(> 50.8 m/s)	NP	55.7
Median SNAP (> 8 μV)	NP	9.1
Sural CV(> 41.9 m/s)	NP	50.5
Sural SNAP(> 7μv)	NP	11.6
**EMG**		
Spontaneous potential	+	-
MUP of large amplitude and increased duration	Extensive	Patrial
**Laboratory examination**		
CK (50–310 U/L)	56	NA
LDH (120-250U/L)	119	NA
**Clinical features**		
Hearing loss	-	-
Pes cavus	-	-
Club foot	-	+
Vocal cord paralysis	-	-
Scoliosis	-	-

*NCS* Nerve conduction study, *CV* Conduction velocity, *Amp* Amplitude, *NP* No potential, *SNAP* Sensory nerve action potential, *MUP* Motor unit potential, *EMG* Electromyogram, *CK* Creatine kinase, *LDH* Lactate dehydrogenase, *NA* Not available; + , present; -, absent

Our case indicated the dynamic and complicated phenotypes of TRPV4-related neuropathies in one family. The phenotypic spectrum of TRPV4-related neuropathies was identified from ventral horn to peripheral nerves, in terms of affected sites (Fig. 3E). Furthermore, a literature review of the clinical characteristics of CMT2C patients [3, 4, 7, 10–17] (Table 2) and SPSMA [3, 9, 13, 18–21] were performed (Table e- 1). We showed that CMT2C might also have other manifestations in addition to nervous system involvement, such as vocal cord paralysis (37/48, 77%), hearing loss (12/42, 29%), and scoliosis (10/37, 27%). The distal and lower limbs are more likely to be affected than the proximal and upper limbs by axon damage. The mutations on the transmembrane region (p.S542Y and p.V620I) are the only cause of skeletal dysplasia leading to short stature [3, 16]. As for SPSMA patients, scapuloperoneal weakness manifesting sloping shoulders, scapular wing and calf muscle atrophy should be noted. Scoliosis and vocal cord paralysis may also be observed. Consistent with patient 2, the clubfoot was also identified in some SPSMA patients.

**Table 2:** Clinical characteristics of CMT2C patients from reported cases

**Case number**	1	2	3	4	5	6	7	8	9	10	
Study	Deng et al. 2020 [10]	Sullivan et al.2015 [12]	Evangelista et al.2015 [13]	Echaniz-Laguna et al.2014 [4]	Landoure et al.2012 [14]	Klein et al. 2011 [11]	Auer-Grumbach et al.2010 [7]	Landoure et al.2010 [14]	Zimoń et al.2010 [3]	Chen et al. 2010 [16]	
**Number** **(male)**	2 (2)	4 (2)	1 (0)	1 (1)	3(1)	4(2)	9	17(6)	1 (0)	3(1)	6(2)
**Mean age at onset**	Childhood	33	40	Childhood	5	39	15	16	6	26/1^a^	7^a^
**Family history**	Incomplete penetrance	AD	De novo	De novo	AD	AD /De novo	AD	AD	De novo	AD	AD
**Mutation site**	R316C R269C	R237G R237L	D62N	R232S	R186Q	R232C R316H	R316C R315W	R269C R269H	V620I	R315W	S542Y
**Sensory involvement**	2/2	4/4	1/1	1/1	3/3	4/4	5/9	16/17	0/1	3/3	2/6
**Proximal UL weakness**	0/2	2/4	1/1	0/1	0/3	0/4	6/7	8/17	0/1	0/3	0/6
**Proximal LL weakness**	0/2	1/4	1/1	0/1	1/3	0/4	NA		0/1	2/3	
**Distal UL weakness**	1/2	3/4	1/1	1/1	3/3	2/4	8/9	17/17	1/1	3/3	4/6
**Distal LL weakness**	2/2	4/4	1/1	1/1	3/3	2/4	9/9		0/1		
**Areflexia**	2/2	2/4	0/1	NA	3/3	4/4	9/9	NA	NA	3/3	6/6
**Scoliosis**	0/2	0/4	0/1	1/1	2/3	0/4	4/7	2/5	1/1	0/3	0/6
**Vocal cord paralysis**	1/2	2/4	0/1	1/1	3/3	2/4	7/9	15/15	0/1	2/3	4/6
**Hearing loss**	1/2	0/4	0/1	0/1	1/3	0/4	NA	10/17	0/1	0/3	0/6
**Pes cavus**	2/2	NA	0/1	1/1	NA	1/4	NA	3/7	NA	NA	1/6
**Other features**				Tremor		Shortness of breath		Bladder incontinence	Short stature	Congenital CN III palsy	Short stature Dolichocephaly

*AD* Autosomal dominant, *UL* Upper limb, *LL* Lower limb, *NA* Not available, *CN* Cranial nerve

^a^Hoarse voice/wheezing

The clinical phenotypes are typically variable and have overlapping features between syndromes. For example, scapulopeoneal muscular atrophy, a typical manifestation of SPSMA can also occur in CMT2C patients. Besides, a clinical phenotype can also evolve into another over disease course, with dHMN potentially shifting to CMT2C later with sensory nerves involvement [16]. It is imperative to closely monitor changes that occur over time.

Regarding the crystal structure model of the human TRPV4 protein, we marked the mutations related to CMT2C and SPSMA with colors (Fig. 3B). These arginine sites were located on the convex face of the domain. All neuropathy-related arginine mutations were in the ARD region, located in the connecting finger loop between inner and outer helices of the ankyrin repeats (Fig. 3C). They mainly involved p.D62 in the N-terminal cytoplasmic domain and p.R186, p.R232, p.R237, p.R269, p.R315, and p.R316 in ARD as well as p.S542 and p.V620 in the transmembrane domains, where skeletal dysplasia was more commonly seen. Neuropathy was more commonly seen with mutations in the ARD region, which was normally responsible for protein–protein interactions [22] (Fig. 3D). Gain-of-function mutations p.R232C, p.R269H, and p.R316H have been noted with increased basal and maximum Ca^2+^ channel activities compared to wild-type TRPV4 [11]. Remarkably, disease-associated TRPV4 mutations that caused a gain-of-function phenotype also abolished PI(4,5)P2 binding to TRPV4 ARD, which can negatively regulate channel activity [23]. Increased channel activity and subsequent Ca^2+^ overload resulted in impaired axonal mitochondrial transport and axonal degeneration [24]. Recent studies have demonstrated that neuropathy mutations but not skeletal dysplasia mutations disrupted TRPV4-RhoA binding and cytoskeletal outgrowth [25]. CMT2C patients with transmembrane region mutation p.S542Y exhibited short stature because of mild metaphyseal dysplasia, while other transmembrane site p.V620I presented a brachyolmia phenotype with scoliosis and short stature. These facts indicated that the transmembrane region mutation was more associated with skeletal dysplasia (Fig. 3D). Two mutations in p.R316, p.R316C and p.R316H, have been reported. The p.R316C mutation was the most common change and has been observed in dHMN [4], SPSMA [4, 7, 18] and CMT2C [7, 10, 11] patients. While, only two cases of p.R316H mutation in *TRPV4* gene have been reported, one of which resulted in CMT2C and the other in dHMN. Both mutations exhibited overall similar clinical characteristics such as weakness of distal limbs and sensory loss. From two previous studies, four out of five p.R316C patients tended to be scapular winging manifesting SPSMA and dHMN, while neither of the two p.R316H patients demonstrated this trait [4, 10]. Additional features like shortness of breath can occur in both mutations. Of note, all seven patients exhibited vocal cord paralysis, which is a higher frequency than observed with other mutation sites. The phenotypic variability of the p.R316 mutation has been documented in the literature. However, only a few reports explained the pathology of nerve biopsy in TRPV4-related neuropathies. Our study presented further pathological details in both semithin sections and ultrastructure examination.

In conclusion, the spectrum of TRPV4-related neuropathies shows considerable phenotypic heterogeneity. Recognizing such striking phenotypic variation is crucial for defining the proper clinical diagnosis. Mutations detected in the TRPV4 ARD region and matched neuropathy features may aid in earlier diagnosis. Extra-nerve manifestations of vocal cord paralysis, scoliosis, and hearing loss should also be highlighted. More studies are needed to elucidate the pathogenic mechanism of TRPV4-related neuropathies, which can aid in identifying appropriate therapies.

## Supplementary Information

**Additional file 1: Table e-1. **The clinical characteristics of *TRPV4* mutations associated with SPSMA.

## Data Availability

There are no associated datasets for this manuscript. All data generated or analyzed during this study are included in this published article. Related queries can be directed to the corresponding author.

## Abbreviations

CMT2C: Charcot-Marie-Tooth disease 2C
SPSMA: Scapuloperoneal spinal muscular atrophy
TRPV4: Transient receptor potential vanilloid type 4
CMAPs: Compound muscle action potentials
SNAPs: Sensory nerve action potentials
EMG: Electromyogram
MUPs: Motor unit potentials
