# Supplementary material for: A TRPV4 mutation caused Charcot-Marie-Tooth disease type 2C with scapuloperoneal muscular atrophy overlap syndrome and scapuloperoneal spinal muscular atrophy in one family: a case report and literature review
Source: BMC Neurol. 2023 Jun 30;23:250. doi: 10.1186/s12883-023-03260-0 (PMC10311707; doi:10.1186/s12883-023-03260-0)
Supplement: Supplementary file 1 — Additional file 1: Table e-1. The clinical characteristics of TRPV4 mutations associated with SPSMA. [file 12883_2023_3260_MOESM1_ESM.docx]

**Method**

The sequenced reads were compared with the reference human genome version (GRCh37/hg19). Nucleotide changes found in aligned reads were pulled and analyzed using the NextGENe software (Version 2.4.2) (SoftGenetics, State College, PA, United States). Online software programs PolyPhen-2, SIFT, PROVEAN, MutationTaster, were used for in silico analysis. Population and literature databases, including gnomAD r2.0.2 (http://gnomad.broadinstitute.org), OMIM (<https://omim.org/>) and ClinVar (<https://www.ncbi.nlm.nih.gov/clinvar>) were used to annotate variants. Variants were classified as “P,”“LP,” “VUS,” “likely benign (LB),” or “Benign (B)” according to the ACMG guidelines.

**Table e-1**

The clinical characteristics of *TRPV4* mutations associated with SPSMA

| **Mutation** | **Author** | **Year** | **Patient number** | | **Label** | | **Phenotype** | | **Symptoms onset** | | **Spinal deformity** | | **Vocal cord paralysis** | | **Additional features** | |
| --- | --- | --- | --- | --- | --- | --- | --- | --- | --- | --- | --- | --- | --- | --- | --- | --- |
| R232C | Zimon et al.[3] | 2010 | 1 | CMT-456.01 | | SPSMA | | Stridor | | Scoliosis | | +, Unilateral | | Hearing loss | |  |
|  | Koutsis et al.[9] | 2015 | 1 | III-2 | | SPSMA and CMT2C | | Gait unsteadiness | | Scoliosis | | +, Partial | | Exertional dyspnea | |  |
|  | Evangelista et al.[13] | 2015 | 1 | Patient 1 | | SPSMA | | Torticollis, difficulty in moving | | Lordosis | | - | | Short stature | |  |
| R269C | Zimon et al.[3] | 2010 | 1 | CMT-858.05 | | SPSMA | | Scapular winging | | - | | +, Asymptomatic | | Pes cavus,  Hammertoes | |  |
|  | Vill et al.[20] | 2015 | 1 | Patient 1 | | SPSMA | | Scapuloperoneal weakness | | - | | - | | Clubfeet | |  |
| R269H | Zimon et al.[3] | 2010 | 2 | CMT-165.01 CMT-165.04 | | SPSMA | | Distal weakness lower limb | | - | | - | | Ankle contracture | |  |
|  | Biasini et al.[19] | 2016 | 2 |  | | SPSMA | | Difficulty in walking, arm elevation | | - | | - | | Clubfeet, metatarsal dysplasia | |  |
| R315W | Zimon et al.[3] | 2010 | 2 | CMT-149.04 | | SPSMA | | Lower limbs pain | | - | | - | | - | |  |
| R316C | Deng et al.*[18] | 2010 | 22 |  | | SPSMA | | Round shoulders | | - | | + | | Breath difficulty | |  |
| +, present; -, absent.  *first reported by Delong et al.(1992)[21] | | | |  | |  | |  | |  | |  | |  | |  |
